# 

**Published:** 1848-06

**Authors:** 


### WESTERN JOURNAL

OF

### MEDICINE AND SURGERY.

### EDITORS.

DANIEL DRAKE, M. D.,

PROFESSOR OF PATHOLOGY AND THE PRACTICE OF MEDICINE
IN THE UNIVERSITY OF LOUISVILLE.

LUNSFORD P. YANDELL, M. D.,

PROFESSOR OF CHEMISTRY AND PHARMACY IN THE SAME

THOMAS W. COLESCOTT, M. D

### INDEX TO VOL. I.

[THIRD SERIES.]

Abortion induced, ..............209
Acupuncture in blemishes of cornea, ........................232
Aconita, Poisoning by..........538
Advance of Asiatic Cholera,....245
Allopathy vs. Homœopathy,....367
American Med. Association, 367,471 547
Anæsthetic Agent, new one,...148
Animal Heat as affected by phthisis.....................80
Antimony, retention of in the living organs,...............258
Antimony, poisoning by,.....76,92
Araneides, Natural History of, 369
Arsenic, Poisoning with.....76, 92
Asthma, (periodical) cured by quinine,..................74

Bandages to the lower extremities in Uterine Hemorrhage, 78
Bite of Spider,................369
Bloomingdale Asylum,.........457
Bowditch's Stethoscopist......519
Browne on Animal torpidity,...240
Burns, ........................5

Cæsarian Section,.............383
Cartwright on Med. Statistics,.185
Catarrh of the Uterus,........103
Cerebral Circulation, disorders of 457 541
Children, Food of............541
Chloroform, 148, 229, 264, 269, 361 455
Chloroform. Death from,......348
Cholera, Asiatic,.....92, 182, 245, 337, 454
Churchill's Midwifery,........458
Cold Water in Fever..........443
Consumption, Naptha in......533
Concours,.....................547
Contribution to Med. Education, 366
Convulsive Affections of Infants.152
Convulsions from Ascaris Lumbricoides, ...................308
Croup, Nitrate of Silver in.....355
Cutaneous Eruptions induced by Medical Substances,...........84
Cyst of the Liver, .............99

Dawson's Reports of Cases, ...285
Death of Christ, physical cause,..26
Death of Dieffenbach, ....184, 354
Death of Liston, ..............184
Death of Prof. Randolph, .....454
Deaths in Medical Class.......273
Denman's Obstetrical Remembrancer.....................520
Diet, Chemical Theory of,......75
Discharge of a Tooth from the Ear, ........................448
Diseases of the U. S. Army on the Rio Grande,.............461
Disorders of the Cerebral Circulation, ......................457
Dublin Dissector..............555
Dubois on Abortion...........209
Dubois ou Uterine Hemorrhage, 287
Dunglison's Prac. of Medicine..404
Dyspeptic Headache, .........441

Eastman's Farriery,..........336
Epidemic Scarlatina,...........461
Epilepsy, treatment of....345, 449
Eschars of Sacrum, ..........230

Etherization in Convulsions, ...277
Etherization in Labor, .........254
Etherization by Sanford,.......302
Etherization by Warren, ......233
Extension of Medical Session in University of Louisville.......276

Faculty of Medicine of Paris,...454
Farcy in Man..................528
Femur, Fractures of,...........94
Fever at Charlotte, Tenn. .....206
Fever treated by cold water,...443
Fever, Yellow, at Charleston,...71
Fever, Ship, at New Orleans,..274
Fractures of the Femur.........94
Food of Children..............541
Fractures, by Jobert.............3
Flood in the Ohio River, .......85

Gangrene, Spontaneous,........387
Glossitis,......................82
Gen. Shields' wound,..........264

Headache of Dyspeptics,..441, 534
Health of Louisville,............92
Hemorrhage from Umbilical cord, 70
Hemorrhage, Uterine,..........387
Hentz on the Araneides,........369
Homœopathy,.............83, 367

Ice in Exhausting Diseases....548
Identity of light, heat, and electricity,..................459
Idiots, Treatment of............92
Infants, Convulsive Affections of 152
Introductory Lectures,......88, 456
Iodine, Tinct. of, in Spina Bifida, 352

Jones on Datura Stramonium..352

Kentucky Institution for the blind,.....................363
Keen's new Instrument for Diagnosis of Tumors,............452

Lallemand on Spermatorrhœa,..330
Larkin's account of Fever,.......206
Light, Heat, and Electricity, identity of.....................459
Lithotomy and Lithrotrity....230
Liston, Death of...............884
Liver not extirpated.............87
Liver, Cyst of,..................99
Eiver, Structure of.............542

Materia Medica, Paines.........518
Matteucci on Living Beings, 459, 521
M'Clellan's Surgery,...........495
Medical Commencement.......453
Medical Classes, ..............453
Medical Department University of Louisville,...............177
Medical School (second) in Louisville,.................178, 275
Med. Statistics, by Cartwright, 182
Medical Quackery—Homœopathy,......................83
Meigs' Letters on Diseases of Females,................. 310
Miller, Dr., Death of..........556
Mosby, Dr. Death of..........556

Naphtha in Consumption.......533
Neuralgia, Prussian Blue in,....231
New Medical School in Louisville,..................178, 275
Nitrate of Silver in Croup,.....355
Nitrate of Silver in Diarrhœa and Dysentery .................450
Nitrate of Silver in Epilepsy,.. 345
Notes on Medical Men and Medical Matters in Paris, 1, 93, 209, 387
Nursing Women, Sore mouth of, 266

Operative Surgery, Velpeau's elements of..............50, 113
Ozone as a cause of Disease,...549

Paine's Materia Medica.........518
Physiological Temperance Society,..........................273
Phthisis, Rostan on, .............11
Poisoning with Arsenic,......76, 92
Poisoning with Tartar Emetic,..23
Proctor on Diseases of U. S. Army on the Rio Grande, .....461
Purpura Hemorrhagica..........21

Quinine in Rheumatism........227

Ranking's Abstract,............365
Rape under influence of Ether..263
Rees's Physiology.............519
Resignation of Dr. Bayless,....460
Retained Placenta,...............62
Rheumatism, Quinine in,.......227

Rupture of Uterus with escape of fœtus into Abdomen......110
Sanford on Use of Ether,......302
San Leopold's Sanitary Establishment,.....................88
Sanitary Survey,..............364
Ship Fever in New Orleans,...274
Simpson on Chloroform,......148
Solly on the Human Brain,....457
Sore Mouth of Nursing Women, 266
Spiders, Natural History &c. of 369
Spina Bifida cured by tinct. Iod. 352
Spontaneous evolution of Fœtus,359
Statististical Medicine, by Dr. Cartwright,................185
Stethoscopist,................519
Stomatitis Materna,.......... 266
Stramonium an emmenagogue..282
Sutton on retained Placenta,....61
Sutton on Encephaloid,.........489

Tartar Emetic, Poisoning by,....23
Taylor on Poisons,............459
Throckmorton, Dr. Death of...555
Torpidity of Animals,......... 240
Tucker's Midwifery,...........335
Transactions of American Medical Association of Philadelphia College of Physicians,....90, 458
Treatment of Croup,...........355
Treatment of Diarrhœa and Dysentery,...................450
Treatment of Dyspeptic Headache,.....................441
Treatment of Epilepsy,....345, 440
Treatment of Idiots,...........92

Umbilical Cord, Hemorrhage from,.....................70
United States' Farriery,........336
Uterus. Rupture of............110
Uterus, Catarrh of,............103

Velpeau's Operative Surgery,50, 113
Vulva, as affected by disease or Pregnancy.................16

Warren on Etherization,......233
Web of Spider, Use of.........369
Whitehead on Abortion and Sterility,.....................458
Wood's Practice of Medicine,..404

Yandell's Letters from Paris. .1 ,93 209, 387

